# Cultural adaptation, validation and evaluation of the psychometric properties of Childbirth Experience Questionnaire version 2.0 in the Spanish context

**DOI:** 10.1186/s12884-024-06400-7

**Published:** 2024-03-19

**Authors:** Elisabet Machín-Martín, Héctor González-de la Torre, Haridian Bordón-Reyes, Julia Jeppesen-Gutiérrez, Alicia Martín-Martínez

**Affiliations:** 1 University of Las Palmas de Gran Canaria, Edificio Ciencias de La Salud, C/Blas Cabrera Felipe S/N, 35016 Las Palmas de Gran Canaria, CP Spain; 2 Department of Obstetrics and Gynaecology, Insular Maternal and Child University Hospital Complex of Gran Canaria-Canary Health Service, Avda Marítima del Sur S/N, 35016 Las Palmas de Gran Canaria, CP Spain; 3 Research Support Unit of Insular Maternal and Child University Hospital Complex of Gran Canaria, Canary Health Service, Avda Marítima del Sur S/N, 35016 Las Palmas de Gran Canaria, CP Spain; 4 Department of Nursing, University of Las Palmas de Gran Canaria, Edificio Ciencias de La Salud, C/Blas Cabrera Felipe S/N, Las Palmas de Gran Canaria, CP 35016 Spain; 5 Multiprofessional Teaching Unit of Obstetrics and Gynaecology of the University Hospital Complex Insular Materno-Infantil of Gran Canaria, Canary Health Service, Avda Marítima del Sur S/N. CP:35016, Las Palmas de Gran Canaria-Canary Islands, Spain

**Keywords:** Obstetrics, labour, Patient satisfaction, Surveys and questionnaires, Validation studies as topic, Childbirth experience questionnaire

## Abstract

**Background:**

Several instruments have been designed to assess the childbirth experience. The Childbirth Experience Questionnaire (CEQ) is one of the most widely used tools. There is an improved version of this instrument, the Childbirth Experience Questionnaire (CEQ 2.0), which has not been adapted or validated for use in Spain. The aim of present study is to adapt the CEQ 2.0 to the Spanish context and evaluate its psychometric properties.

**Methods:**

This research was carried out in 2 stages. In the first stage, a methodological study was carried out in which the instrument was translated and back-translated, content validity was assessed by 10 experts (by calculating Aiken's V coefficient) and face validity was assessed in a sample of 30 postpartum women. In the second stage, a cross-sectional study was carried out to evaluate construct validity by using confirmatory factor analysis, reliability evaluation (internal consistency and temporal stability) and validation by known groups.

**Results:**

In Stage 1, a Spanish version of the CEQ 2.0 (CEQ-E 2.0) was obtained with adequate face and content validity, with Aiken V scores greater than 0.70 for all items. A final sample of 500 women participated in Stage 2 of the study. The fit values for the obtained four-domain model were RMSEA = 0.038 [95% CI: 0.038–0.042], CFI = 0.989 [95% CI: 0.984–0.991], and GFI = 0.990 [95% CI: 0.982–0.991]. The overall Omega and Cronbach's Alpha coefficients were 0.872 [95% CI: 0.850–0.891] and 0.870 [95% CI: 0.849–0.890] respectively. A coefficient of intraclass correlation of 0.824 [95% CI: 0.314–0.936] (p ≤ 0.001) and a concordance coefficient of 0.694 [95% CI: 0.523–0.811] were obtained.

**Conclusions:**

The Spanish version of CEQ 2.0 (CEQ-E 2.0), has adequate psychometric properties and is a valid, useful, and reliable instrument for assessing the childbirth experience in Spanish women.

**Supplementary Information:**

The online version contains supplementary material available at 10.1186/s12884-024-06400-7.

## Background

The childbirth experience is highly significant for women in their maternity journey, and as a result, interest in this topic has surged in recent years [1–6]. In 2018, the World Health Organization (WHO) introduced the concept of the childbirth experience as a focal point for enhancing childbirth care. Understanding a woman's experience during childbirth is deemed crucial to ensuring high-quality care during labour and delivery and improving woman centred outcomes [6]. A positive childbirth experience is defined as a meaningful outcome for all women, meeting their personal and sociocultural expectations, and adhering to minimum requirements for a positive childbirth process [6]. The majority of women aspire to have physiological labour and childbirth experience, aiming for a sense of accomplishment and self-control through active participation in decision-making, even when medical interventions are necessary [2, 6].

It has been established that the childbirth experience is grounded in psychological aspects specific to the woman and her prior expectations [2, 7]. A positive childbirth experience includes effective communication with the healthcare team, the ability to make informed decisions, privacy and a comfortable space with a companion, emotional and psychological support from competent and friendly personnel, and a sense of security [3, 4, 6, 7]. Other crucial factors influencing this perception include shared decision-making and the management of complications through effective coordination with healthcare professionals [5–7]. A positive childbirth experience promotes long-term improvement in the health and well-being of both the mother and the baby by fostering interaction and the establishment of emotional bonds [7, 8]. Furthermore, a recent review revealed an association between a positive birth experience and improved maternal and obstetric outcomes, including cesarean section rates, epidural use rates, episiotomy rates, Apgar scores, and umbilical cord pH of the newborn [9]. It has also been documented that a positive birth experience is directly correlated with reduced labor pain [10, 11].

In contrast, negative experiences during childbirth can increase the incidence of postpartum depression, instil fear of future childbirth, reluctance to have children in the future, influence the choice of caesarean section over vaginal delivery, and lead to poor outcomes in breastfeeding [5, 12–15]. The close relationship between having had a previous negative experience and the fear of childbirth is well-known [15–19].

Given the aforementioned factors, efforts have been made to change childbirth care in recent years, aiming for woman-centred care to enhance their maternity experience. This involves empowering women in decision-making and addressing their biopsychosocial needs related to childbirth [2, 4, 20]. Accumulated evidence has underscored the need to create woman-centred birthing environments that make them feel free, secure, and protected from negative experiences and childbirth fears [19, 21]. Therefore, exploring satisfaction in childbirth is crucial to implement measures that enhance the birthing experience. Additionally, satisfaction with childbirth has been considered a significant obstetric indicator to assess the quality of care provided to women during childbirth [4, 6, 22].

Satisfaction with childbirth is recognized as a complex and multidimensional construct influenced by numerous factors [2, 20, 23, 24]. Various interventions have been proposed to improve maternal satisfaction, such as continuous labour support, pain control, personal self-control, and prenatal classes, among others [2, 5, 25, 26].

Several instruments have been designed to measure childbirth experience and satisfaction [27, 28]. The Childbirth Experience Questionnaire (CEQ), developed in 2010 in Sweden by Dr. Anna Dencker, is one of the most widely used tools for this purpose due to its robust psychometric properties [24]. It has been validated and used in multiple settings and studies [29–40]. Dr. Anna Dencker highlighted the potential for improvement of the instrument, alongside the observation that in the original study, two domains demonstrated weaker performance: Participation and Professional Support [41]. Subsequently, a second version of this instrument, the Childbirth Experience Questionnaire 2.0 (CEQ 2.0), was developed in 2020 to address ceiling effects in some items [41]. This version has also been translated and adapted into various languages and contexts [42–46].

In Spain, the CEQ was validated by Soriano Vidal et al. in 2016 [30], and it is referred to as the Spanish version of the CEQ instrument (CEQ-E). As previously mentioned, the CEQ has been associated with some psychometric issues. Furthermore, recent studies on childbirth experiences worldwide are utilizing the new version of the CEQ, the CEQ 2.0. Since this version has not been validated in Spain, there is consideration for validating it in the Spanish context. This would enable future comparisons of results. Therefore, the objective of this study to adapt the CEQ 2.0 to the Spanish context and evaluate its psychometric properties.

## Methods

This research was conducted in two stages.

- 1st Stage: Methodological study. The translation and transcultural adaptation of the Childbirth Experience Questionnaire 2.0 (CEQ 2.0) were carried out, with an assessment of content validity through expert testing and facial validity, through cognitive interviews and a pilot test in the target population.
- 2nd Stage: Cross-sectional study in a validation sample for the evaluation of construct validity and reliability (internal consistency and temporal stability).

### Stage 1

#### Starting Instrument

The starting instrument was the Childbirth Experience Questionnaire version 2.0 (CEQ 2.0) in its English version [41], provided by its author (Dr A. Dencker).

The original CEQ 2.0 consists of 22 items distributed across four domains: "Own capacity" (items No. 1, 2, 4, 5, 6, 7, 21, and 22), "Professional support" (items No. 11, 13, 14, 15, and 16), "Perceived safety" (items No 3, 17, 18, 19, 20, and 23), and "Participation" (items No. 8, 9, 10, and 12). The first nineteen items are scored on a Likert scale ranging from 1 to 4 points, with some items having reverse scoring. The last three items are numerically ranked on a visual analogue scale from 0 to 100 points, which is then converted to a 4-point scale. Higher scores indicate a more positive childbirth experience. Scores can be obtained for the overall questionnaire by summing the scores of all items and dividing the result by the total number of items or for each domain (summing the scores of the items composing the domain and dividing by the number of items in the domain) [24, 41].

#### Translation procedure

Initially, permission was obtained from the author of the original questionnaire, Dr Anna Dencker, to begin the validation process. Following the principles of Beaton and Guillemin [47], two independent translations of the original CEQ 2.0 from English to Spanish were performed. The translations were carried out by two bilingual translators: a native English-speaking midwife with over 6 years of professional development in England and a certified professional translator and interpreter for the English language. These translations were reviewed by the research team (EMM, HGT, AMM), compared to each other, contradictions were corrected, and a preliminary version integrating both translations was obtained. At this stage, some doubts regarding the interpretation of certain items were discussed with Dr Anna Dencker. The previously completed Spanish version of the CEQ-E [30] was also considered, as some items were identical in both versions. Subsequently, two independent and different bilingual translators from the previous phase performed two back-translations based on this preliminary version. One of them was a midwife who had worked for 15 years in England, and the other was a professional translator unfamiliar with the study topic. These back-translations were evaluated by the research team (EMM, HGT, AMM) and compared to the original questionnaire to ensure equivalence. In addition, the four translators involved were asked to assess the level of difficulty of the four translations as easy, moderate, or difficult. Finally, an external expert (author of the CEQ-E) [30] was asked to evaluate the version obtained in this phase. This resulted in the first Spanish version of the CEQ-E 2.0 (V1 CEQ-E 2.0).

#### Content validity

Content validity was established based on the judgment of 10 experts, consisting of professionals with different profiles. All experts possessed a minimum of a Bachelor's degree and had accrued at least 15 years of professional experience, with seven of them holding a PhD. The criteria for selecting experts included having relevant knowledge and experience in obstetrics and childbirth care, as well as expertise in instrument validation and the development of questionnaires aimed for patients. Five experts had an academic profile and the other five had a more clinical profile. The expert test considered the relevance of each item (whether the item assessed what it intended to evaluate and the importance of the item in relation to the study construct). These criteria were evaluated using Likert scale scores ranging from 1 (Item not relevant) to 4 (Item very relevant). The Content Validity Index for each item (CVI-i) was calculated based on expert scores. The Aiken test was performed, calculating the respective 95% confidence intervals (95% CI) for each item [48]. CVI-i values above 0.70 were considered adequate [49, 50]. The Universal-CVI (UA-CVI) was used to calculate the overall validity of the instrument. This index is the proportion of items on an instrument that achieves a relevance rating of 3 or 4 by all the experts [50].

#### Face validity

A pilot test (pre-test) was conducted in the target population to ensure the questionnaire's comprehensibility and acceptability. This pilot test involved a sample of 30 postpartum women selected through non-probabilistic convenience sampling. Participants were asked to complete the questionnaire on the second postpartum day before hospital discharge and to describe any items they found difficult to answer. They were requested to provide feedback on items in terms of comprehension, relevance, and ambiguity. Cognitive interviews were conducted with 10 of these women, using a debriefing text used in similar studies [51]. Based on the input from these women, a second Spanish version of the CEQ-E 2.0 (V2 CEQ-E 2.0) was obtained.

Finally, this version was evaluated using the INFLESZ scale [52], which measures the comprehensibility of health-related texts for the general population in the Spanish context. The scale classifies texts as Very Difficult (0–40), Somewhat Difficult (40–55), Normal (55–65), Quite Easy (65–80), and Very Easy (80–100) [52].

### Stage 2

#### Design

A cross-sectional study was conducted to obtain a validation sample for the evaluation of construct validity and the calculation of reliability.

#### Setting and study population

The study population consisted of postpartum women whose childbirth took place at the Insular Maternal and Children University Hospital Complex, a tertiary care centre for maternal and child health in the province of Las Palmas, Canary Islands, Spain. In 2022, this centre attended to a total of 3521 births, of which 10% were caesarean sections.

Inclusion criteria considered postpartum women, aged 18 and above, with cephalic or breech presentation, whose last delivery occurred between 37 and 42 weeks of gestation, and whose deliveries were either vaginal or caesarean with labour. Exclusion criteria included women with planned caesarean sections due to obstetric problems (placenta previa, transverse fetal position, breech presentation in women over 40 years of age, two or more previous cesarean sections), women who experienced traumatic psychological incidents during pregnancy or childbirth, women with severe psychiatric problems, women with unexpected admission of the newborn or fetal or neonatal death, and those unable to read/comprehend the questionnaire.

#### Sample size

The original CEQ 2.0 consists of 22 items. According to classical factor analysis theory, there should be at least 10 subjects per item in the instrument to be validated [53]. Based on this, a minimum sample size of 440 women was estimated for factor analysis. This took into account the recommendation of having a minimum of 200 subjects and the potential need for a cross-validation analysis (where the sample is divided into two subsamples to explore the stability of results) [53].

#### Variables studied

The following sociodemographic and obstetric variables were collected: maternal age, education level (no education, primary, secondary, and university), type of labour onset (spontaneous or induced with oxytocin), parity (nulliparous or multiparous), type of delivery (spontaneous cephalic delivery, spontaneous breech delivery, dystocic vaginal breech, caesarean, and forceps), gestational age at delivery, use of increasing oxytocin during labour (yes/no), duration of stay in the delivery room (more or less than 12 h), type of pain relief (epidural pharmacological, intradural pharmacological, no analgesia, non-pharmacological), presence of perineal trauma (yes or no), type of trauma (1st, 2nd, 3rd and 4th degree perineal tear, cervical tear, episiotomy), and breastfeeding at hospital discharge (exclusive breastfeeding, mixed feeding, formula feeding, no breastfeeding).

#### Instrument and data collection system

The data collection process took place from February 28, 2022, to December 23, 2022.

Convenience non-probabilistic sampling was employed. Probabilistic sampling was not considered due to the study's objective, which prioritized securing a large sample size within a reasonable timeframe. Initial recruitment took place in the Immediate Postnatal Unit two hours after delivery. Women who met the inclusion criteria were offered voluntary participation in the study by the midwife of this unit. If they agreed, physical documentation was provided to them by the research team (EMM, JJG, HBR) on the wards at 24 h postpartum.

Women had two options for completing the questionnaire: either physically on-site during admission (between the first and second postpartum day), with the completed questionnaire handed personally to the research team (EMM, JJG, HBR) at discharge, or online (via the Google Forms® platform, following CROSS recommendations [54]). In the latter case, they were notified by telephone beforehand and were given a maximum period of one month after delivery to complete the questionnaire. In this manner, all questionnaires (both those collected in the hospital and online) were consistently completed between days 1 and 30 postpartum.

Obstetric information was extracted from the medical records of each participant by the researchers (EMM, JJP, HBR). For the collection of data on temporal reliability (Test–Retest), 30 women who had physically responded during their admission were randomly selected to complete the questionnaire a second time online.

#### Data analysis and interpretation

A descriptive analysis of the study variables was performed. Qualitative variables were expressed in percentages and frequencies, and quantitative variables were presented as means, standard deviations, and minimum–maximum values. Skewness and kurtosis values were calculated for each item.

#### Construct validity through confirmatory factor analysis

A confirmatory factor analysis (CFA) was conducted based on the initial model proposed for the CEQ 2.0. The appropriateness of the data for factor analysis was assessed using the Kaiser Meyer Olkin index (KMO) and Bartlett's test statistic. Values above 0.75 for KMO and statistically significant values of p ≤ 0.05 for Bartlett's statistic were considered appropriate [53, 55]. A preliminary detection of inappropriate items was performed using Gulliksen's pool based on Relative Difficulty Index (RDI), Item Consistency Index (ICI), and Measure of Sampling Adequacy (MSA) values [56]. A Pearson correlation matrix was utilized, with factor extraction by Robust Unweighted Least Squares (RULS) and oblique PROMIN rotation [53, 55]. Parallel analysis was employed to determine the number of factors to retain, and the consistency of the retained factors was calculated. Bootstrapping was used to calculate 95% confidence intervals for item scores and model measures.

Various indices were used to assess the fit of the factorial solution: Root Mean Square of Residuals (RMSR), Root Mean Square Error of Approximation (RMSEA), Non-Normed Fit Index (NNFI), Comparative Fit Index (CFI), Goodness of Fit Index (GFI) and Adjusted Goodness of Fit Index (AGFI). An RMSR value of 0.05 was considered an acceptable fit, and for RMSEA, values below 0.05 were considered a good fit, while values between 0.05–0.08 were deemed a reasonable fit [52]. NNFI and CFI values of 0.95 or higher, and GFI and AGFI values above 0.90, were considered indicators of a good model fit [52]. Factor consistency was assessed using ORION (Overall Reliability of fully-Informative prior Oblique N-EAP scores) coefficients and the Factor Determinacy Index (FDI) [57].

The Generalized G-H index was calculated to assess the extent to which items reflected a common factor. Values above 0.80 are considered an indicator of a well-defined latent variable that is more likely to remain stable across studies, while low values suggest a poorly defined latent variable that is likely to change across studies [58]. The unidimensionality of the model was evaluated using the Unidimensional Congruence (UniCo), Explained Common Variance (ECV), and Mean of Item Residual Absolute Loadings (MIREAL) indices. UniCo values above 0.95, ECV values above 0.85, and MIREAL values below 0.30 were considered indicative that the data could be essentially considered unidimensional [59].

#### Reliability

Reliability (internal consistency) was evaluated using omega and alpha coefficients. For the calculation of temporal reliability-stability, the intraclass correlation coefficient of a two-factor random effects model and the concordance coefficient were calculated [60]. A Bland–Altman plot was used for the graphical representation of temporal reliability.

#### Validation by known groups and final proposal for CEQ-E 2.0

After obtaining the final structure, an inferential analysis was conducted for validation by known groups. After checking the skewness of distribution of the data obtained using the Kolmogorov–Smirnov test, the non-parametric Mann–Whitney U test was employed for mean comparisons between two groups, and the Kruskall Wallis test was used for mean comparisons among more than two groups, followed by a post hoc test (Dwass-Steel-Critchlow-Fligner) to identify between which groups the differences were found. Statistical significance was set at α ≤ 0.05 for this study. For each association studied the effect size was calculated using Hedges’ g formula, and Kelley’s Epsilon squared measure.

The JAMOVI© v.2.3.24 statistical package was used for the descriptive and inferential analysis of the variables. The FACTOR© Release Version 12.02.01 × 64 bits software was used for factor analysis and model reliability.

#### Ethical considerations

Approval was obtained from the Ethics and Drugs Committee of the Province of Las Palmas (CEIm HUGCDN Code: 2021–353-1). Each participant received a Study Information Sheet and an Informed Consent Form, which they could read, understand, and sign, indicating their voluntary participation in the research. All databases were blinded, with no identifiable participant data.

## Results

### Stage 1

#### Translation procedure

Three of the translators indicated an easy level of translation, and only one of them responded with a medium level. In items No. 8 and No. 16, the meaning of the words "staff" and "team's medical" was discussed because there was a doubt about whether they referred broadly to the entire healthcare team and not just the midwife or doctor. It was decided that they should refer to the overall healthcare team, following the recommendation of the original questionnaire's author. Finally, the external expert approved the final version obtained in this phase, resulting in the V1 CEQ-E 2.0 version.

#### Content validity

The panel of 10 experts comprised 5 midwives, 1 sociologist, 3 obstetricians, and 2 nurses (7 women and 3 men). The professional profile of all the experts can be found in Supplementary Material 1. All items obtained Aiken's V coefficient values above 0.70. Table 1 shows the scores assigned by each expert for each item, along with the values obtained with their respective 95% CI. The UA-CVI obtained was 0.77.

**Table 1:** Experts scores and content validation V Aiken values

Item CEQ-E 2.0	Expert 1	Expert 2	Expert 3	Expert 4	Expert 5	Expert 6	Expert 7	Expert 8	Expert 9	Expert 10	V Aiken Coefficient [95% CI]
1.Labour and birth went as I had expected	4	4	3	4	4	4	4	4	4	4	0.97 [0.83–0.99]
2. I felt strong during labour and birth	4	2	4	4	1	4	4	4	4	4	0.83 [0.66–0.93]
3. I felt scared during labour and birth. R	4	4	4	4	4	4	4	4	4	3	0.97 [0.83–0.99]
4. I felt capable during labour and birth	4	2	4	4	4	4	4	4	4	4	0.90 [0.74–0.97]
5. I was tired during labour and birth. R	4	4	4	4	4	4	4	4	4	4	1.00 [0.89–1.00]
6. I felt happy during labour and birth	4	4	4	2	4	4	4	4	4	4	0.90 [0.74–0.97]
7. I felt that I handled the situation well	4	4	4	4	1	4	4	4	4	4	0.90 [0.74–0.97]
8. I wish the staff had listened to me more during labour and birth R	4	4	4	4	4	4	4	4	4	3	0.97 [0.83–0.99]
9. I took part in decisions regarding my care and treatments as much I wanted	4	4	4	4	3	4	4	4	4	4	0.97 [0.83–0.99]
10. Both my partner and I were treated with warmth and respect	4	4	4	4	4	4	4	4	4	4	1.00 [0.89–1.00]
11. I received the information I needed during labour and birth	4	4	4	4	4	4	4	4	4	4	1.00 [0.89–1.00]
12. I would have preferred the midwife to be more present during labour and birth R	4	4	4	4	4	4	4	4	4	4	1.00 [0.89–1.00]
13. I would have preferred more encouragement from the midwife R	4	4	4	4	4	4	4	4	4	3	0.97 [0.83–0.99]
14. The midwife conveyed an atmosphere of clam	4	4	4	4	4	4	2	4	4	3	0.87 [0.70–0.95]
15. The midwife helped me to find my inner strength	4	4	4	4	4	4	4	4	4	3	0.97 [0.83–0.99]
16. My impression of the team`s medical skill made me feel secure	4	4	4	3	4	4	4	4	4	4	0.97 [0.83–0.99]
17. I have many positive memories from childbirth	4	4	4	4	4	4	4	4	4	4	1.00 [0.89–1.00]
18. I have many negative memories from childbirth R	4	4	4	4	4	4	4	4	4	4	1.00 [0.89–1.00]
19. Some of my memories from childbirth make me feel depressed R	4	4	4	4	4	4	4	4	4	4	1.00 [0.89–1.00]
20. As a whole, how painful did you feel during childbirth? R	4	4	4	4	4	4	4	4	4	4	1.00 [0.89–1.00]
21. As a whole, how much control did you feel you had during childbirth?	4	4	4	4	3	4	4	4	4	3	0.93 [0.79–0.98]
22. As a whole, how secure did you feel during childbirth?	4	4	4	4	4	4	4	4	4	4	1.00 [0.89–1.00]

*R* Item reversed in scoring

#### Face validity

Face validity results showed some aspects in the wording and acceptability of some items for women. Minor wording changes were made to items No. 12 and No. 13 to improve understanding, according to the suggestions made in the cognitive interviews. Ten of the participants (33%) suggested making changes to the response format of items No. 20, No. 21, and No. 22 (addressing pain, self-control, and safety). For this reason, the visual analogue scale (VAS) in these items was changed to a numerical scale from 0 to 10, where 0–3 = 1, 4–5 = 2, 6–7 = 3, and 8–10 = 4 points. The score of 0.67 on the INFLESZ scale indicated a "Quite easy" level of understandability. After these modifications, the V2 CEQ-E 2.0 version was obtained.

### Stage 2

#### Descriptive analysis of the sample and CEQ-E 2.0 items

A total of 597 women were recruited during the study period, of which 500 successfully completed the questionnaire (*n* = 500). The mean age of the participants was 31.94 years (SD = 5.54), with a mean gestational age of 39.6 weeks (SD = 1.17). Regarding the level of education, 40% (200) of the women had university education, 33% (167) had secondary education, 21% (103) had primary education, and only one woman reported having no education. This variable had a 5% (29) rate of missing data.

Based on parity, the sample was divided into 57.8% (289) primiparous women and 42.2% (211) multiparous women. In terms of the type of delivery, 81.6% (408) had a spontaneous cephalic delivery, followed by 9.4% (47) who had a forceps delivery, and 8.0% (40) who had a caesarean section in established labour. Only 1.0% (5) had a breech delivery. Half of the sample was induced (50.4%/252), while 49.6% (248) started labour spontaneously. Epidural analgesia was used by 75% (375) of the women, and oxytocin was used in 57% (285) of them.

A total of 75.6% (378) of the participants experienced labour lasting less than 12 h. The occurrence of some type of perineal trauma in participants reached 72% (360). Only 15% (75) of the women in the selected sample underwent episiotomy. The results for perineal tear variable were 24% (120) for 1st degree tears, 32.2% (161) for 2nd degree tears, and 1.2% (6) for 3rd degree tears. A single cervical tear (0.2%) was detected, and no 4th degree tears occurred. Finally, women with exclusive breastfeeding at discharge were 61.2% (306), those with mixed feeding were 19.8% (99), and those with formula feeding were 14.4% (72).

Descriptive analysis of the items (means and confidence intervals, standard deviation, floor and ceiling scores), as well as skewness and kurtosis values, can be found in Table 2.

**Table 2:** Descriptive analysis of CEQ-E 2.0 items

Items CQE-E 2.0	M [95%CI]^a^	SD^b^	Symmetry	Kurtosis	Floor totally Disagree^c^ N (%)	Ceiling totally Agree^c^ N (%)
1.Labour and birth went as I had expected	2.94 [2.85–3.02]	0.991	-0.591	-0.698	58 (11.6%)	172 (34.4%)
2. I felt strong during labour and birth	3.25 [3.18–3.32]	0.763	-0.732	-0.067	10 (2.0%)	214 (42.8%)
3. I felt scared during labour and birth R	2.65 [2.56–2.75]	1.085	-0.131	-1.284	90 (18.0%)	147 (29.4%)
4. I felt capable during labour and birth	3.28 [3.22–3.35]	0.737	-0.772	0.138	9 (1.8%)	217 (43.4%)
5. I was tired during labour and birth R	2.09 [2.00–2.18]	1.023	0.616	-0.734	169 (33.8%)	71 (14.2%)
6. I felt happy during labour and birth	3.26 [3.18–3.33]	0.861	-0.902	-0.106	20 (4.0%)	246 (49.2%)
7. I felt that I handled the situation well	3.25 [3.18–3.32]	0.789	-0.946	0.561	19 (3.8%)	215 (43.0%)
8. I wish the staff had listened to me more during labour and birth R	3.60 [3.53–3.67]	0.790	-2.050	3.345	22 (4.4%)	375 (75.0%)
9. I took part in decisions regarding my care and treatments as much I wanted	3.51 [3.44–3.59]	0.829	-0.903	5.289	21 (4.2%)	324 (64.8%)
10. Both my partner and I were treated with warmth and respect	3.86 [3.82–3.90]	0.454	-3.904	17.442	5 (1.0%)	445 (89.0%)
11. I received the information I needed during labour and birth	3.76 [3.71–3.81]	0.590	-2.819	8.322	9 (1.8%)	411 (82.2%)
12. I would have preferred the midwife to be more present during labour and birth R	3.55 [3.48–3.63]	0.832	-1.893	2.652	27 (5.4%)	360 (72.0%)
13. I would have preferred more encouragement from the midwife R	3.65 [3.59–3.72]	0.721	-2.297	4.876	18 (3.6%)	381 (76.2%)
14. The midwife conveyed an atmosphere of clam	3.82 [3.77–3.86]	0.497	-3.400	14.524	1 (0.2%)	426 (85.2%)
15. The midwife helped me to find my inner strength	3.71 [3.66–3.76]	0.572	-2.217	5.566	6 (1.2%)	378 (75.6%)
16. My impression of the team`s medical skill made me feel secure	3.74 [3.70–3.79]	0.543	-2.342	5.950	4 (0.8%)	394 (78.8%)
17. I have many positive memories from childbirth	3.51 [3.44–3.58]	0.758	-1.598	2.096	16 (3.2%)	320 (64.0%)
18. I have many negative memories from childbirth R	3.31 [3.24–3.39]	0.897	-1.111	0.216	27 (5.4%)	277 (55.4%)
19. Some of my memories from childbirth make me feel depressed R	3.41 [3.33–3.49]	0.940	-1.453	0.893	38 (7.6%)	327 (65.4%)
20. As a whole, how painful did you feel during childbirth? R	1.71 [1.63–1.79]	0.967	1.181	0.230	284 (56.8%)	43 (8.6%)
21. As a whole, how much control did you feel you had during childbirth?	3.40 [3.33–3.48]	0.894	-1.393	0.910	30 (6.0%)	311 (62.2%)
22. As a whole, how secure did you feel during childbirth?	3.71 [3.65–3.77]	0.663	-2.495	5.923	12 (2.4%)	400 (80.0%)

*R* Item reversed in scoring

^a^Mean [Confidence interval 95%]

^b^Standard Deviation

^c^Only the highest (ceiling) and lowest scores (floor) per question are shown

#### Construct validity through confirmatory factor analysis

A confirmatory factor analysis (CFA) was performed based on the initial four-factor model proposed for CEQ 2.0. The preliminary detection of inappropriate items according to Gulliksen's pool did not find any items that should be removed, based on the values obtained for RDI and ISI. MSA values were above 0.750 for all items (values below 0.500 indicate that the item does not measure the same construct as the rest, advising consideration for removal). The results of this preliminary analysis can be found in Supplementary Material 2.

The KMO values and Bartlett's test statistics indicated an adequate sample fit (KMO = 0.887 [95% CI: 0.835–0.891]; Bartlett = p ≤ 0.001). The four-factor-dimension solution provided an explained variance of 59.43%. The fit values for this model were RMSEA = 0.038 [95% CI: 0.038–0.042], NNFI = 0.982 [95% CI: 0.975–0.986], CFI = 0.989 [95% CI: 0.984–0.991], GFI = 0.990 [95% CI: 0.982–0.991], and AGFI = 0.984 [95% CI: 0.970–0.987], indicating a good model fit. The RMSR was 0.0369 [95% CI: 0.034–0.037] (the expected value of RMSR according to Kelley's criterion for an acceptable model in this case was 0.0448).

Table 3 shows the factorial loadings (after rotation) of the model, with their respective confidence intervals. Based on the obtained factorial loadings, the CFA made changes in the factorial assignment of items No. 1, 5, 8, 10, 14, 15, 16, and 20. All items received loadings above 0.300, except items No. 3 and No. 20. Additionally, three items received factorial loadings in more than one factor (items No. 6, No. 17, and No. 22).

**Table 3:** Rotated loading matrix of CEQ-E 2.0

Items CEQ-E 2.0	Domain 1 Own Capacity	Domain 2 Perceived safety	Domain 3 Participation	Domain 4 Professional support
1.Labour and birth went as I had expected		**0.460** [0.305–0.624]		
2. I felt strong during labour and birth	**0.668**[0.525–0.861]			
3. I felt scared during labour and birth R		**0.279** [0.087–0.449]		
4. I felt capable during labour and birth	**0.841** [0.759–0.978]			
5. I was tired during labour and birth R		**0.416** [0.144–0.632]		
6. I felt happy during labour and birth	0.319 [0.176–0.435]	**0.434** [0.288–0.555]		
7. I felt that I handled the situation well	**0.797** [0.607–0.935]			
8. I wish the staff had listened to me more during labour and birth R				**0.598** [0.399–0.739]
9. I took part in decisions regarding my care and treatments as much I wanted			**0.425** [0.227–0.557]	
10. Both my partner and I were treated with warmth and respect			**0.534** [0.309–0.716]	
11. I received the information I needed during labour and birth			**0.679** [0.507–0.853]	
12. I would have preferred the midwife to be more present during labour and birth R				**0.851** [0.751–0.968]
13. I would have preferred more encouragement from the midwife R				**0.845** [0.614–0.970]
14. The midwife conveyed an atmosphere of clam			**0.687** [0.392–0.875]	
15. The midwife helped me to find my inner strength			**0.689** [0.453–0.847]	
16. My impression of the team`s medical skill made me feel secure			**0.673** [0.481–0.815]	
17. I have many positive memories from childbirth		**0.739** [0.654–0.887]	0.304 [0.182–0.412]	
18. I have many negative memories from childbirth R		**0.836** [0.513–1.023]		
19. Some of my memories from childbirth make me feel depressed R		**0.737** [0.584–0.917]		
20. As a whole, how painful did you feel during childbirth? R		**0.275** [0.038–0.517]		
21. As a whole, how much control did you feel you had during childbirth?	**0.438** [0.035–0.657]			
22. As a whole, how secure did you feel during childbirth?		**0.310** [0.017–0.595]	0.319 [0.110–0.497]	

Loading Values [95% Confidence Interval]

*R* Item reversed in scoring

Item No. 6 ("I felt happy during labour and birth") received loadings in domain 1 of 0.319 and in domain 2 of 0.434. Due to this, it was considered more appropriate to assign it to Domain 2, differently from the original model. Item No. 17 ("I have many positive memories from childbirth") received loadings for domain 2 (0.739) and domain 3 (0.304), and it was deemed appropriate to maintain its assignment to domain 2 according to the original model, as also suggested by the obtained factorial loadings. Finally, item No. 22 ("As a whole, how secure did you feel during childbirth?"), although it received a higher factorial loading for domain 3 (0.319), it was decided to assign it to domain 2 (perceived safety), similar to the original model. This decision was more consistent with the theoretical framework and the similarity of the obtained factorial loadings (0.310 in domain 2).

The values of ORION and FDI for the factors, along with H-latent values, can be found in Table 4. The unidimensionality analysis yielded the following results: UniCo = 0.885 [95% CI: 0.854–0.939], ECV = 0.718 [95% CI: 0.676–0.756], MIREAL = 0.328 [95% CI: 0.305–0.354], supporting the multidimensionality of the model.

**Table 4:** Coefficients ORION, FDI and H-latent by domains

Domain	ORION^a^ [95%CI]	FDI^b^ [95%CI]	H-Latent^c^ [95%CI]
Own capacity	0.843 [0.809–0.877]	0.918 [0.899–0.937]	0.843 [0.806–0.878]
Perceived safety	0.880 [0.833–0.911]	0.938 [0.913–0.955]	0.879 [0.827–0.911]
Participation	0.859 [0.802–0.891]	0.927 [0.896–0.944]	0.859 [0.803–0.893]
Professional support	0.849 [0.769–0.898]	0.922 [0.877–0.948]	0.849 [0.769–0.897]

^a^Overall Reliability of fully-Informative prior Oblique N-EAP scores

^b^Factor Determinacy Index: If factor scores are to be used for individual assessment, FDI values above 0.90 are recommended

^c^High H values (> .80) suggest a well-defined latent variable, which is more likely to be stable across studies, whereas low H values suggest a poorly defined latent variable, which is likely to change across studies

#### Reliability

The overall Omega and Cronbach's Alpha coefficients were 0.872 [95% CI: 0.850–0.891] and 0.870 [95% CI: 0.849–0.890], respectively. The values of both coefficients for each of the domains can be found in Supplementary Material 3.

**Fig. 1:**
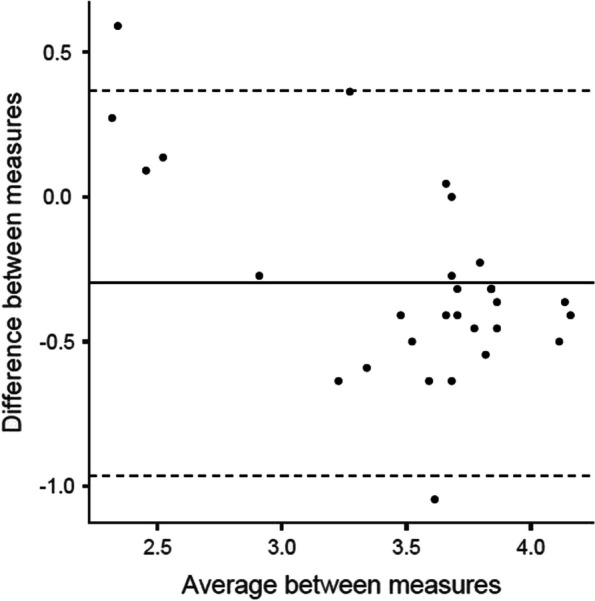
Bland–Altman Plot for test–retest reliability

In the test–retest reliability assessment for measuring temporal stability, a coefficient of intraclass correlation of 0.824 [95% CI: 0.314–0.936] (p ≤ 0.001) and a concordance coefficient of 0.694 [95% CI: 0.523–0.811] were obtained. The Bland–Altman plot (Fig. 1) illustrates the difference between the measurements.

#### Final proposal of CEQ-E 2.0 and validation by known groups

The final proposed model for CEQ-E 2.0 consisted of four domains, named similarly to the original version: Domain 1, ("Own Capacity"), composed of items No 2, 4, 7, and 21; Domain 2, ("Perceived Safety"), composed of items No 1, 3, 5, 6, 17, 18, 19, 20, and 22; Domain 3, ("Participation"), which includes items No 9, 10, 11, 14, 15, and 16; and finally, Domain 4 ("Professional Support"), where items No 8, 12, and 13 are assigned.

The overall mean score was 3.32 (SD = 0.41). By domains, the following means and standard deviations were obtained: Own Capacity 3.30 (SD = 0.59), Perceived Safety 2.95 (SD = 0.56), Participation 3.73 (SD = 0.42), and Professional Support 3.60 (SD = 0.67).

For validation by known groups, the association between some variables and the total scale score and its domains was investigated, also measuring the effect sizes for each inference. Statistically significant differences were found. In Table 5, each of the inferences made for the bivariate variables can be consulted. Statistically significant differences were found for the total scores of CEQ-E 2.0 for the type of labour onset (*p* = 0.010), use of oxytocin (*p* = 0.023), time spent in the delivery room (*p* =  < 0.001), and the presence of perineal trauma (*p* = 0.038). No differences were found in the total score regarding parity and the use of epidural analgesia.

**Table 5:** Validation of Known Groups

	**Domain 1 Own Capacity**	**Domain 2 Perceived safety**	**Domain 3 Participation**	**Domain 4 Professional support**	**CEQ-E 2.0 Total Score**
	**M(SD)**^**a**^	**M(SD)**^**a**^	**M(SD)**^**a**^	**M(SD)**^**a**^	**M(SD)**^**a**^
**Parity**					
Primiparous *n* = 289	3.29 (0.58)	2.91 (0.58)	3.76 (0.38)	3.66 (0.56)	3.31 (0.40)
Multiparous *n* = 211	3.31 (0.61)	3.02 (0.52)	3.69 (0.46)	3.52 (0.77)	3.32 (0.43)
*p*-value^b^	0.526	0.059	0.122	0.295	0.578
Effect size^c^	0.033	0.198	0.168	0.213	0.024
**Type of labour onset**					
Spontaneous *n* = 248	3.33 (0.57)	3.04(0.50)	3.75 (0.42)	3.63 (0.64)	3.37 (0.39)
Induced *n* = 252	3.26 (0.61)	2.87 (0.60)	3.72 (0.41)	3.57 (0.69)	3.27 (0.43)
*p*-value^b^	0.271	0.003*	0.239	0.280	0.010*
Effect size^c^	0.118	0.307	0.072	0.090	0.243
**Use of oxytocin**					
No *n* = 215	3.35 (0.56)	3.05 (0.51)	3.73 (0.43)	3.61 (0.68)	3.37 (0.40)
Yes *n* = 285	3.26 (0.60)	2.88 (0.58)	3.73 (0.40)	3.60 (0.66)	3.28 (0.42)
*p*-value^b^	0.108	0.003*	0.986	0.497	0.023*
Effect size^c^	0.154	0.308	0.000	0.014	0.218
**Time in the delivery room**					
Less than 12 h *n* = 378	3.32 (0.58)	3.03 (0.53)	3.73 (0.43)	3.62 (0.66)	3.35 (0.42)
More than or equal to 12 h *n* = 122	3.24 (0.60)	2.72 (0.58)	3.73 (0.37)	3.56 (0.67)	3.21 (0.39)
*p*-value^b^	0.138	< 0.001*	0.664	0.199	< 0.001*
Effect size^c^	0.136	0.571	0.000	0.090	0.339
**Existence of perineal trauma**					
No *n* = 140	3.28 (0.60)	2.88 (0.62)	3.68 (0.44)	3.52 (0.67)	3.26 (0.44)
Yes *n* = 360	3.30 (0.59)	2.98 (0.53)	3.75 (0.40)	3.63 (0.66)	3.34 (0.40)
*p*-value^2^	0.694	0.119	0.181	0.031*	0.038*
Effect size^3^	0.033	0.179	0.170	0.165	0.194
**Use of epidural analgesia**					
No *n* = 125	3.35 (0.53)	3.03 (0.48)	3.70 (0.47)	3.49 (0.79)	3.33 (0.40)
Yes *n* = 375	3.28 (0.61)	2.93 (0.58)	3.74 (0.40)	3.64 (0.62)	3.31 (0.42)
*p*-value^b^	0.434	0.138	0.383	0.300	0.671
Effect size^c^	0.118	0.179	0.095	0.225	0.048

^a^Mean (Standard Deviation)

^b^Mann-Whitney’s U-test; *statistically significant value

^c^Effect size according to Hedges (Hedges’ g): it considers both groups’ variances and sizes, Values < 0.2 indicate small effects, 0.5 indicates medium effect and 0.8, large effect

Finally, it was determined whether there was a relationship between the variables type of delivery and type of perineal tear in relation to the total score of CEQ-E 2.0, finding a statistically significant association for the type of delivery (*p* =  < 0.001), but not for the type of perineal tear, albeit with a value close to significance (*p* = 0.053). The values of the post hoc test can be found in Supplementary Material 4.

## Discussion

The CEQ, in its two versions, is one of the most widely accepted instruments for assessing women's childbirth experiences [27, 28]. In recent years, researchers have chosen to conduct their studies using version 2, and therefore, despite the validation of CEQ in Spain by Soriano-Vidal FJ et al. (CEQ-E) [30], this research aimed to adapt CEQ 2.0 to the Spanish context and evaluate its psychometric properties. This has already been done in other countries such as Iran or China, where they have adapted and validated versions of CEQ [31, 32, 36] and CEQ 2.0 [43, 45].

The fit obtained for the proposed model of CEQ-E 2.0 achieved suitable values, significantly better than those obtained for CEQ-E, where an RMSEA index of 0.066 was reported for the best of the proposed models [30]. In this study, the obtained CFI and NNF indices were also higher compared to CEQ-E. However, we believe that this can be attributed more to the sample size of Soriano et al.'s study, as they validated it in a sample of 226 women, very close to the recommendation of having a minimum of 200 subjects for conducting a factorial analysis [53], especially when using a polychoric-type matrix, as was their case. Factorial analysis is very sensitive to sample size, and the minimum size to obtain stable factorial solutions is a matter of much debate today [53, 61], with no clear recommendation on the subject. Some authors, like Comrey and Lee, propose indicative quality criteria based on the total sample size (100 = poor, 200 = sufficient, 300 = good, 500 = very good, and 1000 = excellent) [62], while others opt for criteria based on a relationship between the number of cases and the number of variables or based on a relationship between cases and the number of factors [63]. In any case, we consider that 500 women were sufficient to carry out a CFA with guarantees.

The analysis performed according to Gulliksen's pool did not detect items susceptible to removal, as no MSA value was below 0.500 [56]. However, two items obtained factor loads below 0.300: the item "I felt scared during labour and birth", which had a factor load of 0.279 [95% CI: 0.087–0.449], and the item "As a whole, how painful did you feel during childbirth?" with a factor load of 0.275 [95% CI: 0.038–0.517]. Given the confidence intervals calculated for both, it was decided to keep them in the final version, although it is advisable to review the performance of these items in future studies. Both items had also received an excellent rating in content validation by the experts (above 0.80 in both cases).

The item "I felt scared during labour and birth" could be considered a problematic item. The validation study by Lok KYW et al. eliminated it from its final version due to insufficient factor loading [43]. However, discarding items with factor loads below 0.400 is perhaps too strict a criterion, although correct [61]. In the initial study of CEQ, this item received loads above 0.500 (0.51), but compared to the rest of the items, it had one of the lowest loads [24]. This can also be seen in other studies, where this item obtained sufficient but lower factor loads than the rest [31, 45]. Other studies report adequate factor load values for the item [34, 36, 64, 65]. This finding could not be compared in all studies since either they did not conduct factor analysis [32, 40, 42] or did not report factor loads obtained for the items [30, 33, 66].

Fear of childbirth is closely related to satisfaction with childbirth. Rúger-Navarrete et al. established a high correlation between fear of childbirth and the childbirth experience, so that the more fear a woman had, the worse the experience was (*p* = 0.001), precisely using CEQ-E as a measure of the experience [18]. However, in our opinion, they should be considered different constructs, so they should be measured with different instruments. Moreover, there are certain fears and concerns in women that may be directly related to previous experiences [15, 16, 67]. This is a possible explanation for the behaviour of the item.

The other item that obtained insufficient factor loading ("As a whole, how painful did you feel during childbirth?") has been discarded in other studies for this reason. Kalok A et al. eliminated it from the Malay version due to a negative load (-0.822) [34]. In the studies of Zhu X et al. (Chinese version) [31] and Lok KYK et al., it was also discarded [43]. The relationship between pain and negative childbirth experience is well known [2, 11, 68, 69]. However, we must understand that the pain of childbirth is a complex construct, closely related to both external factors (available and provided methods of analgesia to women) [69] and internal factors, related to the woman's coping and perception of control [70, 71]. In different studies with CEQ and CEQ 2.0, multiple pain control methods have been used (also implemented with varied timing), which undoubtedly influenced the functioning of this item. For example, in our sample, a high percentage of women opted for epidural analgesia.

Despite these two items, the fit for the proposed four-domain model was good. Most published models for CEQ and CEQ 2.0 advocate for the existence of 4 factors-domains [30, 31, 34, 36, 41–43, 45], although some studies propose models based on 3 factors-domains [35, 64, 65] or even 6 [33]. Most versions eliminate or make changes to the assignment of items to domains.

What seems clear is the confirmation of the instrument's multidimensionality, something that was already reported in the validation study of CEQ-E, where very poor fit was obtained for the one-factor model (RMSEA 0.200, CFI 0.76, and NNFI 0.74) [30].

The results obtained indicate adequate reliability of the instrument, both concerning internal consistency and temporal stability. The values of Cronbach's alpha and omega coefficients for the total scale are above 0.80, although these values are lower for the domains, similar to what has been reported in other studies with four factors [36, 42, 43]. While most studies conducted to date with CEQ and CEQ 2.0 base the study of internal consistency on the calculation of the Cronbach coefficient, the use of this coefficient as the sole indicator of consistency is quite criticized [72, 73]. Nowadays, the use of the omega coefficient is advised since this coefficient works directly with factor loads, and when the conditions of tau-equivalence (homogeneous covariance between true scores and measurement errors of items) are not met, the resulting alpha coefficient has problems of over or underestimation [73–75]. To date, only the present study and the study by Zhu X et al. [31] have calculated the omega coefficient (0.91 in Zhu X et al.'s study, slightly higher than that obtained in our study).

Additionally, ORION coefficients, the Factor Determinacy Index (FDI), as well as H-latent values per domain, have been calculated. If factor scores are to be used for individual assessment, FDI values above 0.90 and ORION scores above 0.80 are recommended [57]. This, coupled with the fact that H-latent values above 0.80 have been obtained, suggests that there is a well-defined latent variable [58].

Regarding temporal reliability, an adequate intra-class correlation coefficient value was obtained, above 0.8, similar to other studies that have assessed this property in CEQ [32, 33, 35, 36, 40].

Concerning facial validity, CEQ-E 2.0 has shown adequate comprehensibility and acceptability by women, although significant changes were made to items No 20, 21, and 22. In the original CEQ model, these items were answered with a visual analogue scale by marking with an "x" on a line, and the scores from this scale were transformed into values of different ranges (0–40 = 1, 41–60 = 2, 61–80 = 3, and 81–100 = 4) [21]. However, from the analysis conducted after cognitive interviews, it was advised to change the response model to a numerical scale from 0 to 10. Other studies have implemented similar changes to these items [34].

There are evident differences between the populations where different versions of CEQ have been validated and used. Therefore, making comparisons between different scores of domains and the total score among all studies has limited utility in the context of this study. However, a validation by known groups was conducted to check the functioning of CEQ-E 2.0 and compare the results with studies previously conducted with the Spanish version of CEQ (CEQ-E). A study conducted with CEQ-E in the same setting found statistically significant differences in total CEQ-E scores regarding the duration of labour (greater satisfaction in labours lasting less than 12 h), type of delivery (lower satisfaction in instrumental deliveries), and the existence of perineal trauma, not finding differences regarding the use of epidural analgesia or parity [38]. These results are similar to those reported in the present study, which found statistically significant differences for these same variables and found no differences regarding parity or the use of epidural analgesia. Therefore, the functioning of version 2.0 of CEQ-E is similar to CEQ-E in this reference population.

This study has several limitations that must be taken into account. The first is derived from the recruitment system used, as non-probabilistic sampling can affect the accuracy of the results since this system does not ensure collecting all possible cases, and women with negative experiences may not have collaborated in the study. However, this type of sampling has been carried out in all CEQ validation studies.

On the other hand, responses were taken from hospital discharge to the first month after delivery, allowing women to respond at any time within this period. Some authors have pointed out that asking about the childbirth experience shortly after childbirth could have a possible bias in reporting more positive experiences the closer it is to birth [76, 77]. Nevertheless, the results of temporal stability indicate reliability in this regard.

As a final limitation, we can point out that no assessment of convergent/divergent validity with another instrument has been performed, although more psychometric properties have been evaluated compared to CEQ-E.

As the main strength of this work, along with the exhaustive evaluation of internal consistency previously mentioned, is the rigor of the factorial analysis conducted. Most validation studies have used the principal component extraction approach and/or varimax rotation [33, 34, 45, 65], which is as commonly used as it is discouraged today according to current recommendations for factor analysis [53, 55, 61].

This study holds practical implications. In Spain, there remains a paucity of research delving into women's childbirth satisfaction. However, concerning data has emerged regarding Spanish women's perceptions of the care received during childbirth, with notable levels of perceived unjustified interventionism and obstetric violence [78]. Consequently, routine assessment of women's childbirth experiences is warranted to identify negative factors impacting their experience and to implement measures for improvement. Validated instruments should be employed for this purpose, facilitating comparisons across different settings and countries. Given its widespread use and dissemination across numerous countries, we deem the CEQ-E 2.0 as the ideal tool for achieving this objective.

## Conclusions

Although there are several instruments to measure the childbirth experience, the CEQ (in its two versions) is perhaps one of the most widely used, with multiple validations and adaptations. The Spanish version of CEQ 2.0, CEQ-E 2.0, has adequate psychometric properties and is a valid, useful, and reliable instrument for assessing the childbirth experience in Spanish women.

### Supplementary Information

**Supplementary Material 1.**

**Supplementary Material 2.**

**Supplementary Material 3**.

**Supplementary Material 4.**

## Data Availability

No datasets were generated or analysed during the current study.
