# Supplementary material for: Cultural adaptation, validation and evaluation of the psychometric properties of Childbirth Experience Questionnaire version 2.0 in the Spanish context
Source: BMC Pregnancy Childbirth. 2024 Mar 19;24:207. doi: 10.1186/s12884-024-06400-7 (PMC10949694; doi:10.1186/s12884-024-06400-7)
Supplement: Supplementary file 1 — Supplementary Material 1. [file 12884_2024_6400_MOESM1_ESM.docx]

**Professional Profile of the experts in content validation**

| **Expert** | **Gender** | **Professional Field** | **Years of Professional Experience** | **Academic Level** |
| --- | --- | --- | --- | --- |
| 1 | Female | Sociologist. University Professor | 30 | Bachelor’s Degree in Sociology. Ph.D. |
| 2 | Male | Head of Obstetrics and Gynaecology. University Professor | 40 | Bachelor of Medicine and Surgery. Professor of Obstetrics and Gynaecology. Ph.D. |
| 3 | Female | Midwifery manager | 17 | Bachelor’s Degree in Nursing. Bachelor’s Degree in Midwifery |
| 4 | Female | Clinical Midwife | 22 | Bachelor’s Degree in Nursing. Bachelor’s Degree in Midwifery |
| 5 | Female | Clinical Midwife  University Professor | 19 | Bachelor’s Degree in Nursing. Bachelor’s Degree in Midwifery. Ph.D. |
| 6 | Female | Obstetrician-Gynaecologist. Clinical and Management | 16 | Bachelor of Medicine and Surgery. Ph.D. |
| 7 | Male | Clinical Midwife  University Professor | 24 | Bachelor’s Degree in Nursing. Bachelor’s Degree in Midwifery. Ph.D. |
| 8 | Female | Nurse Researcher and Manager  Public Health Nurse | 35 | Bachelor’s Degree in Nursing. |
| 9 | Female | Clinical Midwife | 15 | Bachelor’s Degree in Nursing. Bachelor’s Degree in Midwifery. Ph.D. |
| 10. | Male | Nurse Researcher  University Professor | 31 | Bachelor’s Degree in Nursing. Ph.D. |
