# Supplementary material for: Cultural adaptation, validation and evaluation of the psychometric properties of Childbirth Experience Questionnaire version 2.0 in the Spanish context
Source: BMC Pregnancy Childbirth. 2024 Mar 19;24:207. doi: 10.1186/s12884-024-06400-7 (PMC10949694; doi:10.1186/s12884-024-06400-7)
Supplement: Supplementary file 3 — Supplementary Material 3. [file 12884_2024_6400_MOESM3_ESM.docx]

Omega and Cronbach’s Alpha coefficients by domains.

| **Domain** | **Omega Coefficient [95%CI]** | **Cronbach’s Alpha Coefficient [95%CI]** |
| --- | --- | --- |
| Own capacity | 0.735 [0.684-0.778] | 0.728 [0.674-0.772] |
| Perceived safety | 0.791 [0.756-0.820] | 0.788 [0.756-0.816] |
| Participation | 0.801 [0.744-0.843] | 0.798 [0.737-0.842] |
| Professional support | 0.811 [0.761-0.856] | 0.810 [0.758-0.850] |
