# Supplementary material for: Cultural adaptation, validation and evaluation of the psychometric properties of Childbirth Experience Questionnaire version 2.0 in the Spanish context
Source: BMC Pregnancy Childbirth. 2024 Mar 19;24:207. doi: 10.1186/s12884-024-06400-7 (PMC10949694; doi:10.1186/s12884-024-06400-7)
Supplement: Supplementary file 4 — Supplementary Material 4. [file 12884_2024_6400_MOESM4_ESM.docx]

Validation of Known Groups for type of delivery and type of perineal tear

| **Spontaneous cephalic delivery (n=408)**  **(a)** | **Breech (n=5)**  **(b)** | **Caesarean in established labour (n=40)**  **(c)** | **Forceps**  **(n=47)**  **(d)** |  | **Kruskall-Wallis Test** | **Effect size** | **Post Hoc** |
| --- | --- | --- | --- | --- | --- | --- | --- |
| M(SD) | M(SD) | M(SD) | M(SD) | X^2^ | p Value | Ɛ^2^ | Dwass-Steel-Critchlow-Fligne |
| 3.37 (0.40) | 3.30 (0.59) | 2.92 (0.43) | 3.23(0.35) | 44.8 | <0.001 | 0.0898 | a.c  a,d  c,d |
| M(SD): Mean (Standard deviation)  X^2^= Chi Square  Ɛ^2^= Epsilon squared. Effect size from 0 to 1. with 1 being maximum effect. | | | | | | | |

| **No tear**  **(n=142) (a)** | **1^st^ degree (n=120) (b)** | **2^nd^ degree**  **(n=161) (c)** | **3^rd^ degree**  **(n=6) (d)** | **Cervical tear**  **(n=1) (e)** | **Episiotomy (n=70)**  **(f)** |  | **Kruskall-Wallis Test** | **Effect Size** | **Post Hoc** |
| --- | --- | --- | --- | --- | --- | --- | --- | --- | --- |
| M(SD) | M(SD) | M(SD) | M(SD) | M(SD) | M(SD) | X^2^ | p Value | Ɛ^2^ | Dwass-Steel-Critchlow-Fligne |
| 3.26 (0.44) | 3.34 (0.38) | 3.36 (0.41) | 3.58(0.27) | 3.45 (NaA) | 3.26 (0.43) | 10.9 | 0.053 | 0.0219 | No differences between the groups |
| M(SD): Mean (Standard deviation); NaN=Calculation Not Possible  X^2^=Chi Square  Ɛ^2^= Epsilon squared. Effect size from 0 to 1. with 1 being maximum effect. | | | | | | | | | |
